# Fatal Meningoencephalitis in Child and Isolation of *Naegleria fowleri* from Hot Springs in Costa Rica

**DOI:** 10.3201/eid2102.141576

**Published:** 2015-02

**Authors:** Elizabeth Abrahams-Sandí, Lissette Retana-Moreira, Alfredo Castro-Castillo, María Reyes-Batlle, Jacob Lorenzo-Morales

**Affiliations:** University of Costa Rica, San Pedro, Costa Rica (E. Abrahams-Sandí, L. Retana-Moreira, A. Castro-Castillo);; University of Costa Rica Centro de Investigación en Enfermedades Tropicales, San Pedro (E. Abrahams-Sandí, L. Retana-Moreira, A. Castro-Castillo);; University of La Laguna Institute of Tropical Diseases and Public Health of the Canary Islands, Tenerife, Spain (M. Reyes-Batlle, J. Lorenzo-Morales)

**Keywords:** Naegleria fowleri, hot springs, Costa Rica, primary amebic meningoencephalitis, PAM, Florida, child, encephalitis, ameba

**To the Editor:** Primary amebic meningoencephalitis (PAM) is an acute and fulminant disease caused by *Naegleria fowleri*, an amphizoic ameba belonging to the family *Vahlkampfidae*. About 235 PAM cases have been described worldwide, most in children and immunocompetent young adults (*1*,*2*). The infection occurs through the nose; the ameba enters through the nasal passages and ascends the olfactory nerve until it reaches the olfactory bulb of the central nervous system. The incubation period for PAM ranges from 5 to 7 days, and infection leads to death within a week. Symptom onset is abrupt, with bifrontal or bitemporal headaches, fever, and stiff neck, followed by nausea, vomiting, irritability, and fatigue. The mortality rate is as high as 95%; few cases of survival have been reported (*2*,*3*). 

The epidemiology of most reported cases of PAM indicates an association between aquatic activity and infection. Swimming, free diving, and immersion in hot springs, spas, and warm, freshwater bodies have been related to the acquisition of *N. fowleri* amebae (*3*). To date, most cases have been reported from subtropical or temperate zones, and underreporting in tropical regions has been cited (*2*); in the Americas, PAM has been reported in Venezuela, Brazil, Cuba, Mexico, and the United States. Most infections occur after swimming in water naturally heated by the sun or geothermal water (*2*).

On July 29, 2014, an 11-year old boy, a resident of Florida, USA, was admitted to a hospital for an illness that began after he returned from vacation in La Fortuna, San Carlos, Costa Rica. The infection was fulminant, and the boy died <72 hours after admission. Tests conducted by the hospital confirmed PAM (*4*). The background of the case indicates that the boy spent 1 week in Costa Rica and stayed for 4 days in La Fortuna area. The onset of symptoms occurred 3 days after he left Costa Rica, which is consistent with the incubation period for PAM. Furthermore, the boy’s family stated that in Florida they did not allow him to swim in lakes or rivers because of the known risk of amebal infection (*4*,*5*), which further suggests that the infection may have occurred in Costa Rica. 

The Florida Department of Health was alerted about the case, and personnel from the Centers for Disease Control and Prevention contacted the Costa Rica Ministry of Health to identify the potential source of infection. Water samples from a swimming pool, a river pond, and a hot spring from the resort visited by the boy in La Fortuna were collected and analyzed within 12 hours. The samples were filtered through nitrocellulose membranes with 0.45-µm pore diameter, and the filters were placed over 1.5% non-nutritive agar plates, supplemented with *Escherichia coli* (*6*). Plates were incubated at 35°C for 7 days and observed daily. After 3–4 days of incubation, cysts and trophozoites with morphologic characteristics compatible to *Naegleria* spp. were observed in the samples from the hot spring and the river pond. Cysts were round and 10–12 µm in size, with a *Limax*-type nucleus. Trophozoites showed very active movement, with wide pseudopods of rapid formation. Results of an exflagellation test were positive, and a thermotolerance test showed organism growth at 44°C –45°C (*7*). 

To molecularly characterize the isolate at the species level, we extracted DNA from the culture using the method described by Reyes-Battle et al. (*8*). A specific PCR for *N. fowleri* was performed, and the complete internal transcribed spacer region was amplified as previously described (*1*). The 18S rDNA gene of this free-living ameba was also amplified by using the universal eukaryotic P2 and P3r primer pair (*9*). *N. fowleri* Lee ATCC 30894 DNA was used a positive control in the PCR reactions. The obtained PCR products were purified and sequenced by using a MEGABACE 1000 Automatic Sequencer (Healthcare Biosciences, Barcelona, Spain) in the University of La Laguna Sequencing Service (SEGAI, University of La Laguna). Sequences were obtained twice from both strands and aligned by using MEGA 5.0 software (*10*). Moreover, nucleotide similarity search was performed by BLAST search (http://www.ncbi.nlm.nih.gov/BLAST/) of the sequenced amplicons against ameba species. These analyses revealed 97%–98% homology with other *N. fowleri* strains available in GenBank. The sequence isolated in this case has been deposited in GenBank (accession no. KM658156). 

In summary, this investigation identified an *N. fowleri* ameba in water sources at a resort in Costa Rica that had been visited by a child from the United States who died of PAM as a results of *N. fowleri* infection. These amebas pose a high risk to human health and were found in an area frequented by tourists, which should alert health authorities in Costa Rica of the need for monitoring locations such as this for possible contamination and notifying the public of the risk for infection.
